# Betulinic Acid ω-Triphenylphosphonium Alkyl Esters: Antiproliferative Activities and In Silico Pharmacokinetic Profiles

**DOI:** 10.3390/biomedicines13071539

**Published:** 2025-06-24

**Authors:** Cristian Suárez-Rozas, Claudia Duarte-Salinas, Javier Gajardo-De la Fuente, Paola Salgado-Figueroa, Julio Salas-Norambuena, Bruce K. Cassels, Cristina Theoduloz, José A. Jara, Sebastián Fuentes-Retamal, Paola R. Campodónico, Jorge Soto-Delgado, Mabel Catalán

**Affiliations:** 1 Centro de Química Médica, Instituto de Ciencias e Innovación en Medicina, Facultad de Medicina, Clínica Alemana Universidad del Desarrollo, Santiago 7610658, Chile; c.duartesalinas@uandresbello.edu (C.D.-S.); pcampodonico@udd.cl (P.R.C.); 2 Departamento de Ciencias Químicas, Facultad de Ciencias Exactas, Universidad Andrés Bello, Santiago 8370146, Chile; 3 Faculty of Chemical and Pharmaceutical Sciences, University of Chile, Santiago 8330015, Chile; javier.gdlf@gmail.com (J.G.-D.l.F.); psalgado@ciq.uchile.cl (P.S.-F.); 4 Department of Chemistry, Faculty of Sciences, University of Chile, Santiago 7800003, Chile; julio.salas@umce.cl (J.S.-N.); bcassels@uchile.cl (B.K.C.); 5 Cell Culture Laboratory, Faculty of Health Sciences, University of Talca, Talca 3480094, Chile; ctheodul@utalca.cl; 6 Pharmacology Laboratory, ICOD, Institute for Dental Research, Faculty of Dentistry, University of Chile, Santiago 8330111, Chile; jsandovalj@u.uchile.cl; 7 Escuela de Química y Farmacia, Facultad de Medicina, Universidad Andrés Bello, Santiago 8320000, Chile; sebastianfuentesr@gmail.com; 8 Departamento de Ciencias Químicas, Facultad de Ciencias Exactas, Universidad Andrés Bello, Viña del Mar 2531015, Chile; jorge.soto@unab.cl; 9 Molecular and Clinical Program, Biomedical Science Institute (ICBM), Faculty of Medicine, Universidad de Chile, Santiago 8330111, Chile

**Keywords:** drugs discovery, cancer, antiproliferative activities, betulinic acid derivatives, natural products, synthetic strategy

## Abstract

**Background**: Betulinic acid (**BA**) and some derivatives are well-known antiproliferative compounds. Literature precedents suggest that incorporating triphenylphosphonium (TPP^+^) salts on this triterpenoid scaffold enhances its biological activity. In the present study, we carried out a simple synthesis of C-28 ester derivatives of this triterpenoid conjugated with TPP^+^ bromide salts through 4- to 6-carbon chains via nucleophilic substitution of the corresponding ω-TPP^+^bromoalkanes. Tests for antiproliferative activity in nine cancer cell lines and normal human fibroblasts showed that TPP^+^ incorporation enhanced the potency of **BA** by more than an order of magnitude, up to 100-fold. **BA-C_4_-TPP^+^Br^−^**, with a four-carbon chain separating the TPP^+^ moiety from the **BA**, showed remarkable antiproliferative effects, sometimes more potent than the reference drug (Etoposide). This compound exhibited the strongest mitochondrial uncoupling effect in human cancer cells. No significant LDH release was noted in colorectal carcinoma cells at low micromolar concentrations of **BA-C_4_-TPP^+^Br^−^**, and sub-micromolar concentrations were sufficient for inducing apoptosis. The in silico prediction of pharmacokinetic properties suggested high oral absorption (88%), as well as a non-inhibitor and non-substrate profile vs. cytochrome isoenzymes. These results point to this compound as a promising lead for the development of novel anticancer drugs.

## 1. Introduction

Betulinic acid (**BA**) is a pentacyclic triterpenoid of the lupane skeletal type (3β-hydroxy-lup-20(29)-en-28-oic acid) [1]. This compound is a member of a very large group of natural products, including steroids, derived from squalene by formally acid-promoted cyclization, rearrangement, and degradative reactions [2,3]. Pentacyclic triterpenoids have nourished historically important studies in structure elucidation, reaction mechanism, and stereochemistry, and have displayed multiple bioactivities, among which anti-HIV and antitumor effects stand out [4,5,6,7,8,9,10,11,12].

**BA** can be isolated from materials easily collected as industrial by-products and on our city streets. Many different plant species accumulate it [13], such as, for example, the bark of *Picramnia pentandra* (Simaroubaceae) [14], *Arbutus menziesii* (Ericaceae) [15], or *Ziziphus mauritiana* (Rhamnaceae) [5], but it is particularly accessible as a metabolite of *Platanus* species and hybrids (*P. orientalis* L., *P. occidentalis* L., *P.* × *hispanica* Mill. ex Muenchh. = *P.* × *hybrida* Brot. = *P.* × *acerifolia* Willd.) known as “plane”, “sycamore” (in North America), “London plane”, etc. [16,17]. An alternative source is betulin, the primary alcohol analog of **BA**, which is an abundant metabolite found in the bark of birch (*Betula*) species [18,19]. Different strategies, such as sequential oxidation and reduction reactions [20,21], the formation of suitably protected intermediates for selective oxidation [22,23,24], and promising “single-step” oxidations by treatment with a TEMPO derivative or use of a hypervalent iodine reagent [25,26] have been proposed to access **BA** from betulin [27]. The attractive antiproliferative results of **BA** reported by Farnsworth’s pioneering pharmacognosy group inspired several studies regarding the mechanism of action vs. human cancer cells and the development of novel analogs with improved activity [1,5]. Among the mechanisms proposed for the potent induction of cell death by **BA** is the permeabilization of the mitochondrial membrane with the release of factors such as Smac, cytochrome c, apoptosis-inducing factor (AIF), the activation of caspases, and nuclear fragmentation, leading to intrinsic cell apoptosis, [28]. In spite of its widely recognized antiproliferative and potential anticancer activities, **BA** has some drawbacks, such as its short half-life, which limits its efficacy, and its poor water solubility [29,30].

Mitochondrially targeted drugs such as **BA** represent a new strategy for cancer therapy [31]. Significant differences exist in the structure and function of mitochondria between cancer and normal cells. Extensive studies have shown that there is a large difference in mitochondrial membrane potential (ΔΨm) between the more aggressive kinds of cancer cells (ΔΨm~−220 mV) and normal cells (ΔΨm~−140 mV) [32]. In this context, delocalized lipophilic cations (DLCs) have attracted interest since they offer a successful approach to mitochondrial-targeted chemotherapy due to their capacity to selectively target cancer cells, due to their ΔΨm difference, favoring their accumulation in tumor cell mitochondria and thus inducing tumor toxicity [31]. However, molecules with these characteristics can accumulate (to some extent) in the mitochondria of non-tumor cells. Therefore, a careful selection of the structure and the associated dose for a selective therapeutic effect is necessary [33].

Studies have shown that DLCs can act as selective transporters of functional molecules into mitochondria. Triphenylmethane dyes such as rhodamine B and triphenylphosphonium salts (TPP^+^) are the most widely used moieties for mitochondrial-targeted delivery, as well as the most studied [34,35,36,37,38,39]. Derivatives of lupane triterpenoids conjugated with one or two lipophilic TPP^+^ cations have been prepared, and their cytotoxic, antischistosomal, and bactericidal activities have been described and reviewed [30,40,41,42,43,44]. In vitro pharmacology showed that all of these TPP^+^ salts have stronger antiproliferative activities than their triterpene precursors against small panels of cancer and normal cell lines, although the correlations between additional structural modifications and their biological activities have not been fully established [45].

Inspired by these findings, and noting the absence of any **BA** derivatives modified with a single TPP^+^-alkyl through the C-28 carboxyl group, we carried out a simple synthesis of representative C-28 esters of these TPP^+^ salts with 4- to 6-carbon chains (**BA-C_4_-TPP^+^Br^−^**, **BA-C_5_-TPP^+^Br^−^,** and **BA-C_6_-TPP^+^Br^−^**). While this work was in progress, we were dismayed to find that our compounds had been prepared and assayed against human skin fibroblasts and a couple of human cancer cell lines some time ago [44]. Nevertheless, given the promising published results, we decided to continue with the three compounds we had synthesized, extending the scope of the study to a wider range of cell lines and determining the effects of these TPP^+^ derivatives on oxygen consumption and cell membrane permeability as well as the mechanism of cell death, including the prediction of pharmacokinetic parameters (ADME) by in silico models.

Our synthetic strategy, which differed slightly from that recently published [44], was an extension of the approach used successfully by one of us [46,47]. Instead of adding triphenylphosphine in the final step, we prepared the bromoalkyltriphenylphosphium salts, now under microwave acceleration, to finally form the esters by an uncomplicated nucleophilic substitution reaction with **BA** and triethylamine. **BA-C_4_-TPP^+^Br^−^**, **BA-C_5_-TPP^+^Br^−^**, and **BA-C_6_-TPP^+^Br^−^** were evaluated against a wide panel of human cancer cells (gastric adenocarcinoma, lung cancer, bladder carcinoma, myelocytic leukemia, colorectal and mammary carcinoma) and human lung fibroblasts. In addition, the inhibition of mitochondrial functions was examined. Furthermore, the effect of the most active **BA** derivative (**BA-C_4_-TPP^+^Br^−^**) on the integrity of the cell membrane and death pathways of colorectal carcinoma cells was studied. Pharmacokinetics simulations were undertaken to rationalize the potential application of our compounds in vivo and to foresee the possible development of new generations of Mito-derivatives of **BA**.

## 2. Materials and Methods

### 2.1. Chemistry

All reagents and solvents were commercially available from Sigma-Aldrich (St. Louis, MO, USA) or Merck (Darmstadt, Germany) and were used without further purification. Melting points were uncorrected and were determined using a Reichert Galen III (Vienna, Austria) hot-plate microscope equipped with a DUAL JTEK Dig-Sense thermocouple thermometer. Microwave-assisted organic synthesis was performed in an Anton Paar GmbH (Graz, Austria) Monowave 400 microwave reactor. High-resolution mass spectra were recorded for MeOH solutions on a MALDI-TOF Microflex spectrometer (Bruker Daltonics Inc., Billerica, MA, USA) in the positive-ion detection mode. ^1^H and ^13^C NMR spectra were recorded at 400 MHz and 100 MHz, respectively, on a Bruker Avance III HD-400 spectrometer, using CDCl_3_ or DMSO-*d*_6_ as solvent. The chemical shifts were reported as δ (ppm) from TMS for ^1^H NMR and relative to the DMSO-*d*_6_ resonance (39.5 ppm) for ^13^C NMR. Coupling constants (*J*) are given in Hz. Purities of the compounds subjected to biological testing were >98% in every case (quantitative ^1^H NMR or HPLC).

#### 2.1.1. Isolation and Purification of Betulinic Acid (BA)

The air-dried peeling stem bark of “London plane” (*Platanus* × *hispanica* Mill. Ex Münchh.) was broken into small pieces (about 1 cm square), with 10 g placed in a 250 mL round-bottomed flask, along with MeOH (125 mL) and a few small boiling chips, before being refluxed for twenty minutes. At the end of this period, the hot MeOH solution was filtered through a Büchner funnel, the bark was washed with additional hot MeOH (3 × 20 mL), it was filtered, and the methanol extracts were combined. The solution was concentrated to 50 mL, and the precipitate formed at room temperature was collected by filtration (510 mg). The solid was purified by flash column chromatography on silica gel using CHCl_3_-MeOH (2:8) to afford **BA** (183 mg). **BA** was recrystallized twice in MeOH to afford 167 mg. The spectral data (^1^H- and ^13^C-NMR) were in accordance with those published [48,49,50,51,52]. Mp: 316 °C; ^1^H NMR (400 MHz, DMSO-*d*_6_) δ 12.09 (s, 1H, H_acid_), 4.69 (brs, 1H, H-29a), 4.56 (brs, 1H, H-29b), 2.96 (m, 1H, CH-19), 1.64 (s, 3H, CH_3_-30), 0.92 (s, 3H, CH_3_-23); 0.86 (s, 6H, CH_3_-24, CH_3_-27); 0.76 (s, 3H, CH_3_-25); 0.64 (s, 3H, CH_3_-26). ^13^C NMR (101 MHz, DMSO-*d*_6_) δ 177.23 (C-28), 150.31 (C-20), 109.63 (C-29), 76.80 (C-3), 55.42 (C-5), 54.91, 50.52 (C-9), 49.94, 48.55 (C-19), 46.62 (C-18), 42.01, 38.50 (C-1), 38.27 (C-13), 37.60 (C-22), 36.63, 34.32 (C-7), 32.16 (C-16), 30.56 (C-15), 29.70 (C-21), 27.98 (C-23), 27.39 (C-2), 25.50 (C-12), 20.85 (C-11), 19.37 (C-30), 18.28 (C-6), 16.12 (C-26), 16.03 (C-25), 15.94, 15.74 (C-24), 14.39 (C-27).

#### 2.1.2. General Procedure for the Synthesis of Triphenylphosphonium Bromide Salts (1–3)

Briefly, a G10 microwave vial was charged with a solution of the respective dibromoalkane (2.0 mmol) in CH_3_CN (2.0 mL) and triphenylphosphine (1.0 mmol). The flask was heated as fast as possible (program selected) to 200 °C for a reaction time of 90 s (the maximum power output was 850 W, and the actual power of the microwave energy was dynamically adjusted to maintain the specified temperature). Then, the reaction mixture was cooled to ambient temperature and concentrated to dryness under vacuum to give a pale-yellow oil. The crude mixture was purified by flash column chromatography on silica gel using AcOEt and then AcOEt-MeOH (4:1) to give the pure product.

(4-Bromobutyl)triphenylphosphonium bromide (**1**)

White solid (93%). ^1^H NMR (400 MHz, DMSO-*d*_6_): δ 7.86−7.65 (m, 15H, ArH), 3.91–3.84 (m, 2H, CH_2_), 2.54 (t, *J* = 6.0 Hz, 2H, CH_2_), 2.30–2.24 (m, 2H, CH_2_), 1.83–1.78 (m, 2H, CH_2_). The spectroscopic data are in good agreement with those reported in the reference [53].

(5-Bromopentyl)triphenylphosphonium bromide (**2**)

Pale-yellow sticky oil (92%). ^1^H NMR (400 MHz, DMSO-*d*_6_): δ 7.81–7.64 (m, 15H, ArH), 3.89–3.82 (m, 2H, CH_2_), 2.51 (t, *J* = 6.1 Hz, 2H, CH_2_), 2.26–2.14 (m, 2H, CH_2_), 1.79–1.71 (m, 2H, CH_2_), 1.59–1.51 (m, 2H, CH_2_). The spectroscopic data are in good agreement with those reported in the reference [54].

(6-Bromohexyl)triphenylphosphonium bromide (**3**)

Pale-yellow sticky oil (95%). ^1^H NMR (400 MHz, DMSO-*d*_6_): δ 7.79–7.61 (m, 15H, ArH), 3.88–3.81 (m, 2H, CH_2_), 2.49 (t, *J* = 6.1 Hz, 2H, CH_2_), 2.21–2.10 (m, 2H, CH_2_), 1.76–1.67 (m, 2H, CH_2_), 1.61–1.45 (m, 4H, CH_2_). The spectroscopic data are in good agreement with those reported in the reference [53].

#### 2.1.3. General Procedure for the Synthesis of Betulinic Acid Esters with Triphenylphosphonium Bromide Salts (**BA-C_4_-TPP^+^Br^−^**_,_ **BA-C_5_-TPP^+^Br^−^** and **BA-C_6_-TPP^+^Br^−^**)

A solution of **BA** (1.0 g, 2.18 mmol) in CH_3_CN (70 mL) was cooled to 0 °C. Then, triethylamine (304 μL, 2.18 mmol) was added to the cooled solution and stirred for 10 min. Subsequently, the appropriate triphenylphosphonium bromide salt (2.18 mmol) was added, and the reaction mixture was stirred and refluxed for 72 h (the reaction progress was monitored by TLC). The reaction mixture was cooled to room temperature, the precipitate formed was filtered off, and the solvent was removed in vacuo to give a pale-yellow oily residue, which was pre-purified by flash column chromatography on silica gel eluting with hexane-AcOEt (4:1) and then MeOH. Finally, the partially purified residue was again purified by column chromatography on silica gel using CH_3_CN-DCM (4:1) to give the respective esters.

4-Triphenylphosphoniobutyl 3β-hydroxylup-20(29)-en-28-oate bromide (**BA-C_4_-TPP^+^Br^−^**)

White solid. Yield 76%. Mp: 130–131 °C. ^1^H-NMR (400 MHz, DMSO-*d*_6_) δ 7.99–7.48 (m, 15H, ArH), 4.64 (s, 1H, H-29a), 4.55 (s, 1H, H-29b), 4.29 (brs, 1H, H-3), 4.05 (t, *J* = 5.6 Hz, 2H, CH_2_-O), 3.69 (t, *J* = 13.3 Hz, 2H, CH_2_-P), 2.95 (t, *J* = 7.9 Hz, 1H, H-19), 2.17– 0.90 (m, 29H, remaining betulinic acid scaffold and (CH_2_)_2_ fragment of linker), 1.61, 0.89, 0.86, 0.75, 0.73, 0.64 (s, 3H, 18H, CH_3_-23, CH_3_-24, CH_3_-25, CH_3_-26, CH_3_-27, CH_3_-30). ^13^C NMR (101 MHz, DMSO-*d*_6_): δ 176.0 (C-28), 150.4 (C-20), 135.1 (d, *J_c,p_*: 2.8 Hz, C-Ph), 133.7 (d, *J_c,p_*: 10.1 Hz, C-Ph), 130.6 (d, *J_c,p_*: 12.5 Hz, C-Ph), 118.2 (d, *J_c,p_*: 85.9 Hz, C-Ph), 109.8 (C-29), 78.9 (C-3), 62.8, 56.5, 55.3 (C-5), 50.5 (C-9), 49.4 (C-19), 47.0 (C-18), 46.3, 42.4, 40.7, 38.9, 38.7 (C-1), 38.2 (C-13), 37.2 (C-22), 34.3 (C-7), 32.1 (C-16), 30.6 (C-15), 29.6 (C-21), 28.1 (C-23), 27.4 (C-2), 25.5 (C-12), 22.6, 22.1, 20.9 (C-11), 19.6, 19.3 (C-30), 18.3 (C-6), 16.2 (C-26), 16.0 (C-25), 15.5 (C-24), 14.7 (C-27). Found, *m*/*z*: 773.4948 [M-Br]^+^. Calculated, *m*/*z*: 773.50626 (C_52_H_70_ O_3_P). The spectroscopic data are in good agreement with those reported in the reference [44].

5-Triphenylphosphoniopentyl 3β-hydroxylup-20(29)-en-28-oate bromide (**BA-C_5_-TPP^+^Br^−^**)

White solid. Yield 87%. Mp: 195–196 °C. ^1^H NMR (400 MHz, DMSO-*d*_6_): δ 7.93–7.73 (m, 15 H, ArH), 4.67 (s, 1H, H-29a), 4.55 (s, 1H, H-29b), 4.29 (brs, 1H, H-3), 3.97 (t, *J*: 5.7 Hz, 2H, CH_2_-P), 3.61 (t, *J*: 5.7 Hz, 2H, CH_2_-O), 2.94 (t, 2.96 (m, 1H, H-19), 2.21– 0.95 (m, 31H, remaining betulinic acid scaffold and (CH_2_)_2_ fragment of linker) 1.63, 1.20, 0.92, 0.86, 0.75, 0.64 (s, 3H, 18H, CH_3_-23, CH_3_-24, CH_3_-25, CH_3_-26, CH_3_-27, CH_3_-30).^13^C NMR (101 MHz, DMSO-*d*_6_): δ 176.1 (C-28), 150.6 (C-20), 135.0 (d, *J_c,p_*: 2.8 Hz, C-Ph), 133.7 (d, *J_c,p_*: 10.0 Hz, C-Ph), 130.6 (d, *J_c,p_*: 12.5 Hz, C-Ph), 118.2 (d, *J_c,p_*: 85.9 Hz, C-Ph), 109.6 (C-29), 79.0 (C-3), 63.2, 56.3, 55.3 (C-5), 50.5 (C-9), 49.3 (C-19), 46.9 (C-18), 46.3, 42.5, 40.7. 38.9, 38.7 (C-1), 38.3 (C-13), 37.2 (C-22), 37.1, 34.3 (C-7), 32.2 (C-16), 30.6 (C-15), 29.7 (C-21), 28.0 (C-23), 27.4 (C-2), 25.5 (C-12), 23.0, 22.1, 20.9 (C-11), 19.4, 19.3 (C-30), 18.3 (C-6), 16.1 (C-26), 16.1 (C-25), 15.4 (C-24), 14.7 (C-27). Found, *m*/*z*: 787.5103 [M-Br]^+^. Calculated, *m*/*z*: 787.52191 (C_53_H_72_O_3_P). The spectroscopic data are in good agreement with those reported in the reference [44].

6-Triphenylphosphoniohexyl 3β-hydroxylup-20(29)-en-28-oate bromide (**BA-C_6_-TPP^+^Br^−^**)

White solid. Yield 83%. Mp: 205–207 °C. ^1^H NMR (400 MHz, DMSO-*d*_6_): δ 7.94–7.73 (m, 15 H, ArH), 4.66 (s, 1H, H-29a), 4.55 (s, 1H, H-29b), 4.30 (brs, 1H, H-3), 4.00 (t, *J*: 5.7 Hz, 2H, CH_2_-P), 3.77 (t, *J*: 5.7 Hz, 2H, CH_2_-O), 2.97 (t, 2.96 (m, 1H, H-19). 2.22–1.24 (m, 31H, remaining betulinic acid scaffold and (CH_2_)_2_ fragment of linker), 1.64, 1.18, 0.92, 0.86, 0.75, 0.64 (s, 3H, 18H, CH_3_-23, CH_3_-24, CH_3_-25, CH_3_-26, CH_3_-27, CH_3_-30). ^13^C NMR (101 MHz, DMSO-*d*_6_) δ 176.2 (C-28), 150.5 (C-20), 135.0 (d, *J_c,p_*: 2.8 Hz, C-Ph), 133.7 (d, *J_c,p_*: 10.0 Hz, C-Ph), 130.6 (d, *J_c,p_*: 12.6 Hz, C-Ph), 118.3 (d, *J_c,p_*: 85.9 Hz, C-Ph), 109.7 (C-29), 79.0 (C-3), 63.8, 56.3, 55.4 (C-5), 50.5 (C-9), 49.3 (C-19), 46.9 (C-18), 46.2, 42.5, 40.7, 38.9, 38.7 (C-1), 38.4 (C-13), 37.2 (C-22), 37.1, 34.3 (C-7), 32.5, 32.2 (C-16), 30.6 (C-15), 29.7 (C-21), 28.0 (C-23), 27.4 (C-2), 25.5 (C-12), 23.0, 22.4, 20.9 (C-11), 19.4, 19.3 (C-30), 18.3 (C-6), 16.1 (C-26), 16.1 (C-25), 15.4 (C-24), 14.7 (C-27). Found, *m*/*z*: 801.5322 [M-Br]^+^. Calculated, *m*/*z*: 801.53756 (C_54_H_74_O_3_P). The spectroscopic data are in good agreement with those reported in the reference [44].

### 2.2. Biological Activity

#### 2.2.1. Cell Culture

Human cell lines were obtained from the American Type Culture Collection (ATCC, Manassas, VA, USA). Normal lung MRC-5 fibroblasts (ATCC^®^ CCL-171™), SK-MES-1 lung cancer cells (ATCC^®^ HTB-58™), and J82 bladder carcinoma cells (ATCC^®^ HTB-1™) were grown as monolayers in Eagle’s minimum essential medium (MEM) with Earle’s salts, 2 mM L-glutamine, and 1.5 g/L sodium bicarbonate. Gastric epithelial AGS cells (ATCC^®^ CRL-1739™) were grown as monolayers in Ham’s F-12 medium containing 1 mM L-glutamine and 1.5 g/L sodium bicarbonate. Promyelocytic leukemia HL-60 (ATCC^®^ CCL-240™), colorectal adenocarcinoma HCT-15 (ATCC^®^ CCL-225™), metastatic colorectal COLO205 (ATCC^®^ CCL-222™), and breast cancer AU565 (ATCC^®^ CRL-2351™, ER^−^-HER2/neu^+^) cells were grown in suspension in an RPMI-1640 medium containing 1 mM sodium pyruvate and 2.0 g/L sodium bicarbonate. Human breast cancer MCF-7 (ATCC^®^ HTB-22™, ER^+^-HER2/neu**^−^**) and MDA-MB-231 (ATCC^®^ HTB-26™, ER**^−^**-HER2/neu**^−^**) cells were grown as monolayers in DMEM medium. All media were supplemented with 10% heat-inactivated fetal bovine serum (HI-FBS), 100 IU/mL penicillin G, and 100 µg/mL streptomycin. Cells were maintained at 37 °C in a humidified incubator with 5% CO_2_.

#### 2.2.2. Cell Viability Assay

MRC-5, SK-MES-1, J82, and AGS cells were seeded at a density of 5 × 10^4^ cells/mL in 96-well plates. COLO205, HCT-15, MCF-7, MDA-MB-231, and AU565 cells were seeded at 1 × 10^5^ cells/mL, while HL-60 cells were plated at 3 × 10^5^ cells/mL. A volume of 100 µL of the cell suspension was added to each well. After 24 h, the compounds were added in increasing concentrations (ranging from 0 to 100 µM), and cells were incubated for 24, 48, or 72 h. The compounds were dissolved in DMSO and diluted in complete medium to a final concentration of 0.5% DMSO. Cells treated with 0.5% DMSO in complete medium were used as viability controls (100%). Etoposide (98% purity, Sigma-Aldrich, St. Louis, MO, USA) was used as the reference compound. Each concentration was tested in quadruplicate, and each experiment was performed twice using independent cell preparations. At the end of the incubation period, cell viability was determined using the MTT reduction assay [55]. The results were transformed into percentages relative to controls, and IC_50_ values were obtained graphically from the dose–response curves. Results are presented as IC_50_ values ± standard deviation (SD).

#### 2.2.3. Oxygen Consumption Assay

Oxygen consumption of the tumor cells was measured polarographically using a Clark-type electrode (Yellow Springs Instruments, Dayton, OH, USA) placed horizontally in a cell of 0.6 mL volume at 25 °C, coupled to a YSI monitor (model 53). The electrode signal was recorded in a computer connected to a DI-148U module with a USB interface. Data were acquired using the Windaq Acquisition Waveform Recorder software v3.36 (DataQ Instruments, Akron, OH, USA). The test medium used contained 150 mM NaCl, 5 mM KCl, and 1 mM Tris-HCl, and pH 7.4. 5 mM glutamine was added with the cells and test compounds. Oxygen consumption was recorded when the cell aliquots (6 × 10^6^ cells) were added. Oligomycin (1.5 μg/mL) was added as an inhibitor of the F_o_ subunit of ATP synthase prior to the incorporation of the compounds under study. FCCP (carbonyl cyanide-*p*-trifluoromethoxyphenylhydrazone, 50 nM) was used prior to the addition of test compounds as an internal control to calibrate the electrode system and confirm the maximum uncoupling capacity of the cells.

#### 2.2.4. LDH Release Assay

COLO205 and HCT-15 cells were cultured at 2 × 10^4^ cells/well in 96-well plates and incubated for 24 h. Afterwards, **BA-C_4_-TPP^+^Br^−^** was added in increasing concentrations (0.5, 1, 2.5, and 5 µM) for 24 h. Finally, the supernatant of each well was transferred to a new plate (10 µL/well), and the LDH reaction mix was added (100 µL/well). The plate was incubated for 20 min at room temperature, and the absorbance of each well was measured using a Varioskan^®^ Flash plate reader (Thermo Scientific, Waltham, MA, USA) at 450 nm. All values are expressed as a percentage of LDH activity relative to cells incubated with Cell Lysis solution (provided in the kit) for 24 h [56].

#### 2.2.5. Annexin V/Propidium Iodide Double Staining Assay

The type of cell death induced by the **BA-C_4_-TPP^+^Br^−^** was assessed by flow cytometry, using Annexin V/propidium iodide (AV/PI) double staining, following the instructions of the Annexin V-FITC Apoptosis Detection Kit (Abcam, Cambridge, UK). Cells were cultured at 5 × 10^4^ cells/well in 24-well plates and incubated overnight. Afterwards, cells were treated with increasing concentrations of **BA-C_4_-TPP^+^Br^−^** (1, 2.5, 5, or 10 µM for COLO205 and HCT-15 cells) for 24 h. Finally, cells were trypsinized, resuspended in annexin V binding buffer 1X, adding AV and PI, and incubated for 5 min at room temperature, protected from light. Control corresponds to cells incubated with DMSO 1%. Fluorescence of each probe was assessed by flow cytometry (FACSAria^®^III, BD Biosciences, Franklin Lakes, NJ, USA). Wavelengths used were 488_Ex_/530_Em_ nm for AV-FITC and 488_Ex_/575_Em_ nm for PI. Results are expressed as total apoptotic cells (percentage of AV+/PI− and AV+/PI+ cells), necrotic cells (percentage of AV-/PI+ cells), and live cells (percentage of AV-/PI− cells). Cyflogic software (non-commercial 6th version, CyFlo Ltd., Turku, Finland) was used for data processing [56].

#### 2.2.6. Statistics

All results are expressed as mean ± standard deviation (SD) of at least three independent experiments. The comparison between experimental groups and controls was performed by one-way ANOVA with Bonferroni post-test, using GraphPad Prism 5.0 software. A *p*-value < 0.05 was considered the minimum significance level. IC_50_ values were obtained using the same software, from data fitted to non-linear dose–response regressions, each performed in triplicate. The comparison between different dose–response curves was performed by two-way ANOVA with Bonferroni post-test, using GraphPad Prism 5.0 software. A *p*-value < 0.05 was considered the minimum significance level.

## 3. Results and Discussion

### 3.1. Chemistry

**Scheme 1:**
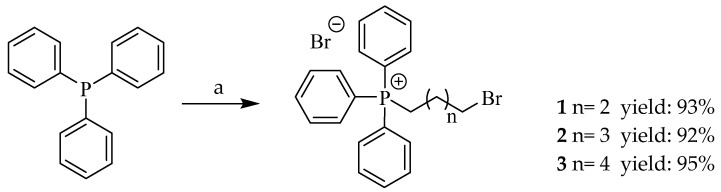
Synthesis of ω-bromo-alkyltriphenylphosphonium bromides **1**–**3**. Reagents and conditions: (a) MW, apropiate dibromoalkane, CH_3_CN, 90 sec.

Delocalized lipophilic cation drugs (DLCs) (**BA-C_4_-TPP^+^Br^−^**, **BA-C_5_-TPP^+^Br^−^**, and **BA-C_6_-TPP^+^Br^−^**) derived from **BA** via a two-step convergent synthesis were prepared. First, the lipophilic chain conjugated to TPP^+^ was synthesized from triphenylphosphine and linear α,ω-dibromoalkanes (1,4-dibromobutane, 1,5-dibromopentane, and 1,6-dibromohexane). Several methods for the preparation of TPP^+^ bromide salts have been reported in the literature [46,47,57,58,59]; however, these approaches often require long reaction times, high temperatures, and/or the use of dibromoalkanes as solvents. In this work, we devised an alternative strategy for the synthesis of ω-bromoalkyltriphenylphosphonium bromides (**1**–**3**) under microwave-assisted conditions. Briefly, the intermediates **1**–**3** were obtained by reacting triphenylphosphine with dibromoalkanes in CH_3_CN as solvent. The mixture was irradiated with microwaves for 90 s at 200 °C, affording the respective products in good yields (92–95%) without noticeable formation of by-products (Scheme 1), demonstrating higher efficiency.

**Scheme 2:**
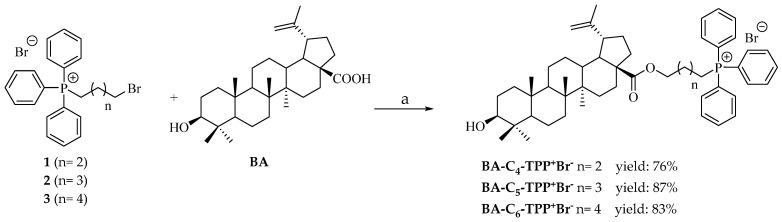
Preparation of C-28 ester derivatives of betulinic acid. Reagents and conditions: (a) CH_3_CN, (CH_3_CH_2_)_3_N, 60 °C, 72 h.

**BA** was isolated from the methanolic extract of the bark of “London plane” (*Platanus* × *hispanica* Mill. Ex Münchh.) collected on the streets of Santiago, Chile. The final step in the synthesis of the mitochondrial-targeted derivatives involved a nucleophilic substitution reaction between the C-28 carboxyl group of **BA** and the primary halide carbon of the corresponding triphenylphosphonium bromides (Scheme 2).

In an attempt to reduce reaction times, we also tested microwave irradiation at 120, 150, 170, and 200 °C for different durations; however, only decomposition products were obtained. In addition, the synthetic approach used to obtain the ω-triphenylphosphonium alkyl esters derived from **BA** resulted in overall yields of 71–80%, an improvement compared to the 63% yield reported by Tsepaeva and co-workers for all three structures [44]. Thus, this methodology proves to be an efficient strategy for the preparation of this class of molecules.

### 3.2. Biological Activity

#### 3.2.1. In Vitro Cell Growth Inhibition

In order to identify the structure with the highest antiproliferative activity and selectivity over non-neoplastic cells, as well as to attempt a preliminary structure-activity relationship (SAR), we first used the MTT reduction assay in four human cancer cell lines (AGS: gastric adeno-carcinoma cells; SK-MES-1: lung cancer cells; J82: bladder carcinoma cells; and HL-60: promyelocytic leukemia cells), including a normal human fibroblast cell line (MRC-5). The concentrations of the compounds inhibiting cell growth by 50% (IC_50_ values) were obtained by adjusting the dose–response curves to a sigmoidal model (Table 1). Additionally, etoposide was used as a control reference.

According to the cellular viability results shown in Table 1, all compounds presented micromolar IC_50_ values in the different cancer cell lines evaluated. **BA** showed moderate activity and selectivity over normal fibroblasts, being particularly active against gastric adenocarcinoma cells. Linking **BA** to the TPP^+^ cations through C-28 indeed led to markedly stronger antiproliferative potency, as seen previously with breast cancer (MCF-7), prostate adenocarcinoma (PC-3), and human skin fibroblast (HSF) cell cultures [44]. Upon closer analysis of the biological data reported by those authors, no clear trend could be discerned between the length of the alkyl chain linking the TPP^+^ moiety to the **BA** scaffold and the antiproliferative activity against the cell lines evaluated. While their results do not show a linear association between the number of methylene units and potency, it is worth noting that the **BA-C_5_-TPP^+^Br^−^** derivative displayed some of the lower EC_50_ values across both neoplastic cell lines, although the difference was not impressive. In contrast to our findings, the **BA-C_4_-TPP^+^Br^-^** analog exhibited weaker activity than its C_5_ and C_6_ counterparts (**BA-C_5_-TPP^+^Br^−^** and **BA-C_6_-TPP^+^Br^−^**, respectively). Interestingly, that earlier paper also reported a progressive increase in cytotoxicity toward skin fibroblasts (HSF cells) as the alkyl linker length increased (but only from C_4_ to C_6_), a trend not observed in our study using lung fibroblasts (MRC-5 cells) [44]. Additionally, the IC_50_ values were mostly lower than those for etoposide, falling within the sub-micromolar to 1–2 μM range.

Given the significant antiproliferative effect and the increased activity observed in the evaluated neoplastic cell lines compared to the non-modified core structure, we decided to further investigate the toxicological profile of **BA** derivatives. To this end, different treatment regimens (varying incubation times) were evaluated in a broader panel of tumor cells, including colorectal cancer cell lines (Table 2) and breast cancer cell lines (Table 3), to obtain preliminary insights into potential cytotoxic or cytostatic profiles [60]. These exploratory results lay the groundwork for future investigations involving complementary assays, which will be necessary to unequivocally determine the nature of the observed antiproliferative effects.

Initially, the antiproliferative activity of TPP^+^ **BA** analogs was assessed against human colorectal carcinoma (HCT-15) and metastatic colorectal cancer (COLO205) cells at three incubation times (24, 48, and 72 h; Table 2). Upon analysis, the TPP^+^ conjugated derivatives exhibited a moderate increase in activity against COLO205 cells, in contrast to **BA** (more active against HCT-15 cells). Generally, the Mito derivatives reached their maximum effect at 48 h of treatment for both cell lines. However, **BA-C_4_-TPP^+^Br^−^** continued increasing its activity against COLO205 cells, reaching an IC_50_ value of 0.13 μM at 72 h, which suggests a potentially cytotoxic and antiproliferative profile against this cell line. Nevertheless, further studies involving more robust and specific methodologies are required to confirm this preliminary evidence and to define the nature of the antiproliferative response. In this same context, **BA-C_4_-TPP^+^Br^−^** emerged as the most potent derivative, suggesting that increasing the alkyl chain length linking the TPP^+^ fragment to **BA** is not beneficial for activity. Interestingly, although **BA-C_6_-TPP^+^Br^−^** was not the most active compound against the evaluated colorectal cancer cells, it exhibited the most pronounced increase in activity between 24 and 48 h.

These compounds were tested against three human mammary carcinoma cell lines: MCF-7, MDA-MB-231, and AU565, incubated for two different treatment times (Table 3), considering that the maximum effect of these compounds was observed after 48 h of exposure in colorectal cancer cell lines (Table 2). Upon analysis, we found that all three **BA-TPP^+^Br^−^** compounds exert antiproliferative effects in the low micromolar concentration range against cells with diverse tumor characteristics, without showing significant differences among the IC_50_ values. Consistent with our initial observations (Table 2), **BA-C_4_-TPP^+^Br^−^** was identified as the most potent compound across all evaluated breast cancer cell lines, further confirming the dependence of activity on the length of the alkyl linker between TPP^+^ and **BA**. Additionally, it is important to highlight the therapeutic potential of these molecules, particularly their activity against the triple-negative metastatic cell line (MDA-MB-231), which lacks the expression of hormone receptors and the human epidermal growth factor receptor-2 (HER2). Clinically, this subtype is the most aggressive among breast tumors, often showing limited response and efficacy to conventional chemotherapeutic agents [61,62].

All these experiments showed that the prepared TPP^+^ and **BA** conjugates exerted strong antiproliferative activities in nine human cancer cell lines. Unfortunately, the increase in potency (IC_50_ values) did not result in a general increase in the selectivity of the compounds against the tumor cell lines compared to normal fibroblasts (MRC-5 cell line). However, **BA-C_4_-TPP^+^Br^−^** showed an increase of up to approximately 7-fold (HCT-15 cells at 72 h), 15-fold (AU565 cells at 48 h), and even 264-fold (COLO205 cells at 72 h) compared to the non-modified **BA** scaffold, including IC_50_ values lower than those of the reference drug used (etoposide). Thus, the most potent compound, **BA-C_4_-TPP^+^Br^−^**, was selected for further studies to elucidate its mechanism of action and the type of induced cell death.

#### 3.2.2. Oxygen Consumption

**Figure 1:**
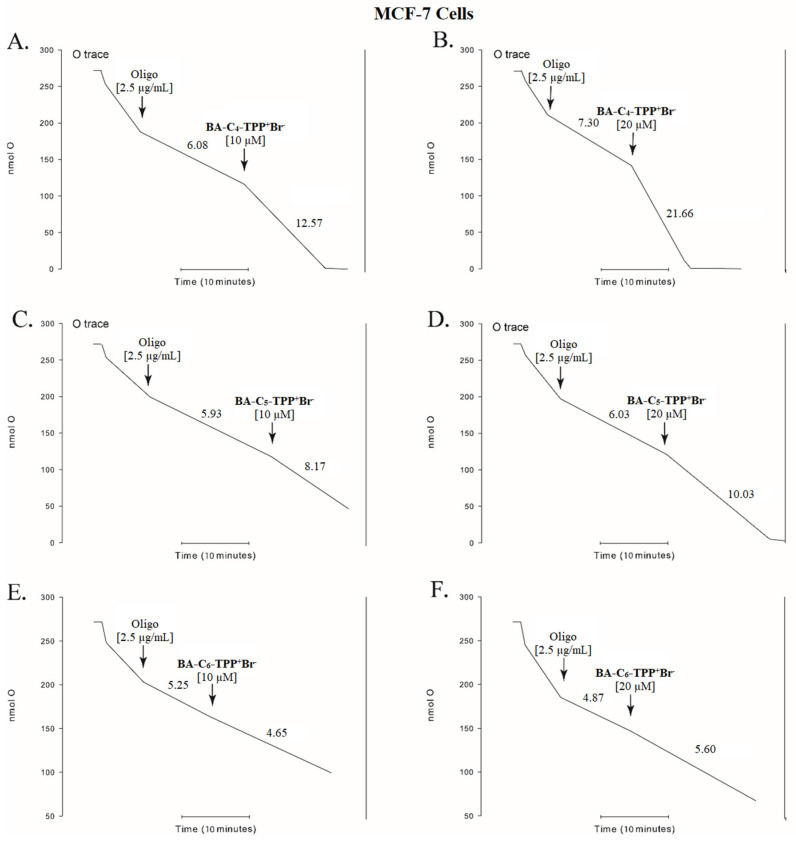
Effect on mitochondrial O_2_ consumption of the MCF-7 cell line at two concentrations of **BA-C_4_-TPP^+^Br^−^** (**A**,**B**), **BA-C_5_-TPP^+^Br^−^** (**C**,**D**), and **BA-C_6_-TPP^+^Br^−^** (**E**,**F**). The number above the trace represents oxygen consumption measured in ng-atoms O/min. (mean, n = 3). The lines were fitted using SigmaPlot 12 software.

To test the hypothesis that the antiproliferative effects of these TPP^+^ derivatives are primarily due to the inhibition of mitochondrial function, we evaluated the oxygen consumption of human mammary cancer cells (MCF-7 and AU565) in the presence of these compounds. Cellular respiration was evaluated with the inhibition of ATP synthase with oligomycin, the uncoupling of the electron transport chain with FCCP, and the effects of the compounds. Figure 1A,B show the cellular respiration rates and the effect of **BA-C_4_-TPP^+^Br^−^** uncoupling the electron transport. The result showed that the uncoupling effect is concentration-dependent, as demonstrated by an oxygen consumption rate of 12.57 nmol O_2_ min^−1^ at 10 µM **BA-C_4_-TPP^+^Br^−^** and 21.66 nmol O_2_ min^−1^ at 20 µM, as illustrated in Figure 1A and Figure 1B, respectively. To further investigate whether the antiproliferative activity of these compounds is intrinsically related to their mitochondrial effects, we also evaluated their structural analogs with alkyl linker chains of five and six carbon atoms. In this regard, **BA-C_5_-TPP^+^Br^−^** also exhibits a concentration-dependent uncoupling effect, though less pronounced than that of **BA-C_4_-TPP^+^Br^−^**, as indicated by a milder increase in the slope of the curve (Figure 1C,D). On the other hand, Figure 1E,F show that if **BA-C_6_-TPP^+^Br^−^** has any uncoupling effect, it is not seen at the tested concentrations, as its addition does not elicit an appreciable change in the slope. The length of the aliphatic chain or the nature of the lipophilic cation induces distinct responses in mitochondrial function, as evidenced by our results. In a previous study conducted by one of the co-authors of this manuscript, it was demonstrated that aliphatic chains containing ten to twelve carbon atoms, when linked to the triphenylphosphonium moiety, could induce a more pronounced mitochondrial uncoupling effect; however, this response was observed in the context of a different bioactive scaffold [46]. On the other hand, it has been previously described that short aliphatic chains of **BA**, when attached to other lipophilic cations such as F16 (a nitrogen-based lipophilic cation), are more effective in triggering mitochondrial activity [63]. Altogether, these findings suggest a strong correlation between the antiproliferative potency of this class of compounds and their mitochondrial uncoupling effect, particularly for derivatives bearing shorter alkyl chains. However, the activity observed for **BA-C_6_-TPP^+^Br^−^**, despite the absence of significant uncoupling, leads us to hypothesize that additional mechanisms may contribute to its cytotoxic profile.

**Figure 2:**
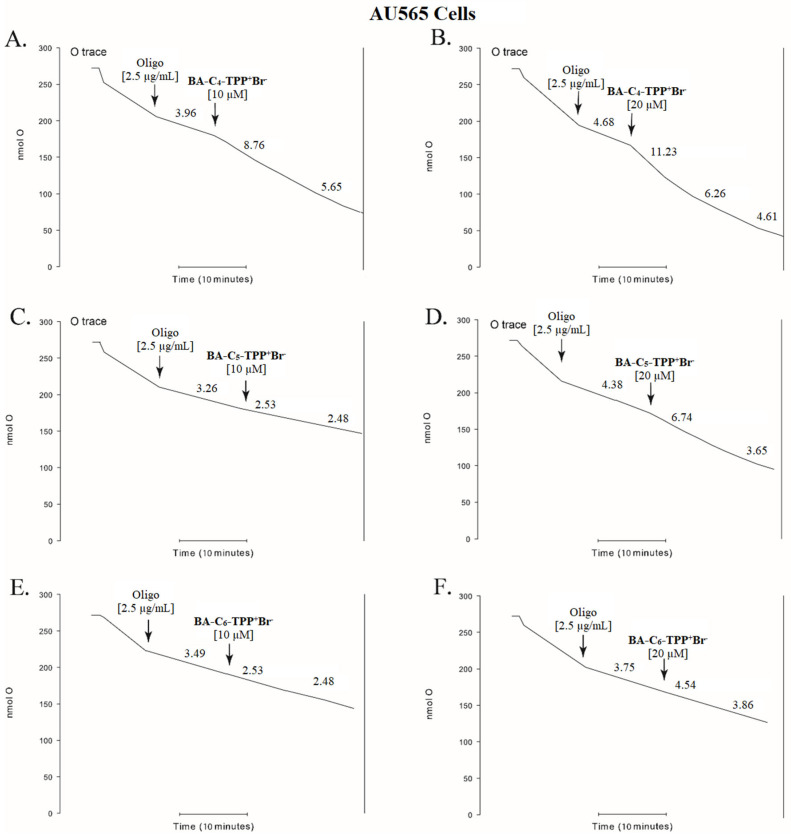
Effect of **BA-C_4_-TPP^+^Br^−^** (**A**,**B**), **BA-C_5_-TPP^+^Br^−^** (**C**,**D**), and **BA-C_6_-TPP^+^Br^−^** (**E**,**F**) on the mitochondrial O_2_ consumption of the AU565 cell line at two concentrations. The number above the trace represents oxygen consumption measured in ng-atoms O/min. (mean, n = 3). The lines were fitted using SigmaPlot 12 software.

Similarly, Figure 2 shows the effects of the three previously tested compounds on an ER^−^ and HER2/neu^+^ mammary carcinoma cell line. In this case, the uncoupling effect is less pronounced than in the MCF-7 cell line. Nevertheless, the correlation between potency (IC_50_ value) and the uncoupling effect remains, as **BA-C_4_-TPP^+^Br^−^** is both the most potent derivative in the antiproliferative assay and the strongest uncoupler in AU565 cells.

#### 3.2.3. Effect of **BA-C_4_-TPP^+^Br^−^** on Membrane Permeability in Human Colorectal Carcinoma Cells

In recent years, some of us have shown a strong interest in developing new therapeutic alternatives for the treatment of colorectal neoplasms (particularly at metastatic stages), which is the third most common cancer worldwide and the second leading cause of cancer-related mortality. In addition, colorectal cancer is one of the most aggressive malignancies, with limited pharmacological treatment options once the metastatic stage is reached [64,65]. Considering these factors, and motivated by the remarkable potency and pronounced uncoupling effect of **BA-C_4_-TPP^+^Br^−^** on the evaluated neoplastic cells, we decided to further investigate its impact on membrane integrity by assessing the leakage of cellular components, as well as its ability to induce apoptosis at varying doses (cell death pathway). LDH release was assayed to evaluate necrotic cell death, which may result from membrane rupture associated with cytotoxic stress. Given the amphipathic nature of the TPP^+^ conjugates, we considered it relevant to explore whether their interaction with membranes could lead to a loss of integrity at the plasma membrane level. To this end, COLO205 and HCT-15 cells were treated with **BA-C_4_-TPP^+^Br^−^** at increasing concentrations (0.5–5 μM) for 24 h of incubation.

**Figure 3:**
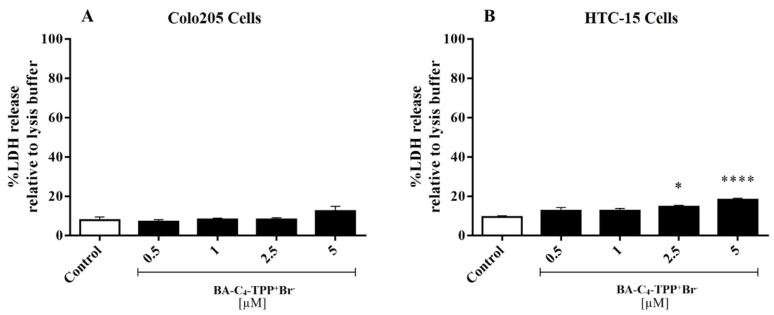
LDH release induced by **BA-C_4_-TPP^+^Br^−^**. COLO205 (**A**) and HTC-15 (**B**) cells were incubated with 0.5, 1, 2.5, and 5 µM of **BA-C_4_-TPP^+^Br^−^** for 24 h. LDH release was assessed by means of the LDH-Cytotoxicity Assay Kit II, as described in the Section 2. Control represents cells incubated only with vehicle (DMSO), and all values are expressed as percentage LDH release relative to that observed after the addition of Cell Lysis solution (mean ± SD, n = 3). *: significant difference compared to control (one-way ANOVA with Bonferroni post-test; *p* < 0.05), ****: *p* < 0.0001.

Figure 3 shows no significant LDH release from COLO205 cells, even at the highest concentration. However, in the HCT-15 cell line, LDH release was evident beginning at 2.5 μM (18%). This test suggests that there is no significant necrotic effect of **BA-C_4_-TPP^+^Br^−^** in human metastatic colorectal cancer cells (COLO205). However, in non-metastatic cells (HCT-15), release increased to double the control (DMSO) value, suggesting some increase in permeability of the cell membrane and loss of cytosolic contents.

#### 3.2.4. Analysis of the Induction of Apoptosis by **BA-C_4_-TPP^+^Br^−^** in Human Colorectal Carcinoma Cells

**Figure 4:**
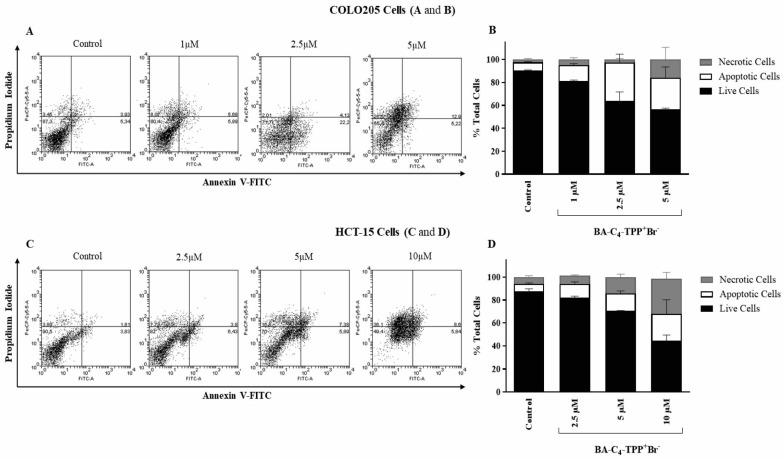
Apoptosis induction on COLO205 and HCT-15 cells incubated with **BA-C_4_-TPP^+^Br^−^**. COLO205 human colorectal carcinoma cells and HCT-15 human colorectal adenocarcinoma cells were incubated with 1, 2.5, 5, or 10 μM **BA-C_4_-TPP^+^Br^−^** for 24 h. Representative dot plots are presented for treatment at 1, 2.5, and 5 μM in COLO205 cells (**A**) and 2.5, 5, and 10 μM in HCT-15 cells (**C**). Quantification of dot plots for (**B**) COLO205 cells and (**D**) HCT-15 cells was performed using Cyflogic software (version 1.2.1). Alive cells (black bars) were defined as annexin V negative/propidium iodide negative, apoptotic cells (white bars) as the grouping of annexin V positive/propidium iodide negative (early apoptosis) and annexin V positive/propidium iodide positive cells (late apoptosis), and necrotic cells (gray bars) as annexin V negative/propidium iodide positive. Control columns represent cells incubated only with vehicle, and results are expressed as a percentage of total cells (mean ± SD, n = 3).

To examine whether the observed cytotoxicity of **BA-C_4_-TPP^+^Br^−^** was due to apoptosis induction, cells were double-stained with annexin V-FITC and propidium iodide probes and evaluated by flow cytometry. COLO205 cells were incubated with 1–5 μM concentrations and HCT-15 cells at 2.5–10 μM concentrations for 24 h before reading. Figure 4 shows the dose-dependence of the activity in both lines of human colorectal carcinoma, with non-metastatic cells (HCT-15) exhibiting lower sensitivity consistent with the cell viability results found in the MTT assay (Table 2). The results show that **BA-C_4_-TPP^+^Br^−^** induced close to 14% apoptosis at the lowest concentration evaluated (1 μM) in COLO205 cells. The 2.5-fold increase in the concentration of the compound increased the apoptotic death pathway, reaching an induction close to 34%. However, at 5 μM, a slight increase in toxicity through a necrotic pathway of cell death may be noted (from 5% in the initial concentration to 15% in the maximum concentration tested). In the HCT-15 cell line, a moderate change was observed in the cell death pathway from apoptotic to necrotic, consistent with the results obtained in the LDH release assay (Figure 4). For the lowest concentration tested (2.5 μM), the induction levels of apoptosis and necrosis were 12% and 7%, respectively. When the concentration of **BA-C_4_-TPP^+^Br^−^** was increased 2- and 4-fold, necrotic cell death (close to 30% at 10 μM) increased more than the apoptotic pathway.

### 3.3. In Silico Pharmacokinetic Profile of BA and Related Compounds

The SwissADME website is a convenient tool for the prediction of drug likeness profiles of compounds only tested in vitro [66,67]. Its use with **BA** and **BA-C_n_-TPP^+^Br^−^** provided different results based on Lipinski’s [68] and Veber’s rules [69] (Table 4). 3-*O*-(3′,3′-Dimethylsuccinyl)-**BA** (**4**), which reached phase II-B in clinical trials (Bevirimat; Clinical trial.gov Identifier NCT00511368) as a potential anti-HIV drug [70,71], was included in order to have a good reference point concerning the derivatives prepared in this study.

The criteria for acceptance of candidates as orally active drugs based on Lipinski’s rule [68] are MlogP ≤ 4.15, MW ≤ 500 Da, ∑HBD ≤ 5, and ∑O + N ≤ 10), while Veber’s rule considers rotatable bonds (≤10) and TPSA (≤140 Å^2^), the latter inversely proportional to %ABS [69].

The in silico pharmacokinetics results indicated that **BA** violates only one of the rules established by Lipinski and Veber, due to its predicted Log P_o/w_ values (MlogP > 4.15), which largely explains the low bioavailability (plasma concentrations lower than 1% of the administered oral dose) reported for naturally occurring pentacyclic triterpenes [72]. However, a study on the intraperitoneal (IP) administration of **BA** in CD-1 mice yielded more encouraging results [73]. In the case of **4** (Bevirimat), this hemisynthetic derivative of **BA** presents two violations of Lipinski’s rule (MlogP > 4.15 and MW > 500 Da). However, pharmacokinetic clinical trials following oral administration demonstrated rapid oral absorption, a long half-life, and good tolerance at all studied doses in both healthy individuals and patients with HIV [74,75]. Therefore, permeability and cell membrane penetration may not be strictly determined by the size and lipophilicity of **BA** derivatives. In the case of DLCs derived from **BA (BA-C_4_-TPP^+^Br^−^**, **BA-C_5_-TPP^+^Br^−^**, and **BA-C_6_TPP^+^Br^−^**), these compounds violate two of Lipinski’s rules (MlogP > 4.15 and MW > 500 Da) and one of Veber’s rules (rotatable bonds > 10). However, two critical aspects must be considered regarding these criteria. First, the calculation of lipophilicity for DLCs has a major limitation: the in silico method applied to these structures does not account for ionic compounds, treating them as neutral molecules. As a result, the prediction model provided partition coefficients higher than expected. Second, the inclusion of an aliphatic fraction bound to the TPP^+^ cation within the pharmacophore (at least 400 Da) leads to an MW exceeding the threshold established by the “Rule of Five.” Nonetheless, given the characteristics of these mitochondriotropic agents (their large hydrophobic surface and positively charged phosphorus atom surrounded by three hydrophobic phenyl groups), they exhibit low activation energy for movement across the hydrophobic membrane core, making them promising candidates for in vivo studies [76,77]. Moreover, DLCs derived from **BA** exhibited an oral absorption rate above 88%, indicating good permeability, absorption, and transport across biological membranes. The predicted metabolism analysis indicated that each candidate is neither an inhibitor nor a substrate of cytochrome isoenzymes (CYP), particularly CYP2D6 and CYP1A2. These results suggest that the compounds are promising drug candidates since they do not interfere with cytochrome enzyme activity. Disruption or inhibition of cytochrome enzymes, which play a key role in metabolism, can often lead to undesirable systemic side effects [78,79]. Supporting these considerations, there is growing evidence that bioactive compounds conjugated to the TPP^+^ fragment have been successfully translated to preclinical studies in animal models, demonstrating favorable outcomes in the in vivo systems used. In fact, one of the authors of this study has previously demonstrated the preclinical application of such mitochondria-targeted compounds in syngeneic mouse models, yielding promising preliminary efficacy results [46]. More closely related to the present work, Ye and collaborators confirmed the in vivo activity of a TPP^+^-conjugated derivative of betulin (a compound structurally related to **BA**) in a zebrafish xenograft model. In that study, the authors observed a significantly reduced migration of neoplastic cells, and the compound effectively inhibited both the proliferation and metastasis of K562 cells (a chronic myeloid leukemia cell line) at low concentrations, showing enhanced activity compared to the non-conjugated betulin scaffold [30]. Taken together, these in silico predictions and preclinical literature reports strongly encourage future investigations aimed at testing this class of molecules in in vivo models.

## 4. Conclusions

In recent years, bioactive compounds of natural origin conjugated with a TPP^+^ moiety have shown promise as a strategy for developing new chemotherapeutic agents targeting cancer. In the present study, a series of delocalized lipophilic cations derived from **BA** were synthesized. The cell viability results for these compounds (**BA-C_4_-TPP^+^Br^−^**, **BA-C_5_-TPP^+^Br^−^**, and **BA-C_6_-TPP^+^Br^−^**) in human cancer cell lines (including gastric adenocarcinoma (AGS), lung cancer (SK-MES-1), bladder carcinoma (J82), myelocytic leukemia (HL-60), colorectal carcinoma (HCT-15 and COLO205), and mammary carcinoma (MCF-7, MDA-MB-231, and AU565)) showed a significant increase in potency (over 100-fold), with IC_50_ values comparable to, and in some cases lower than, those of etoposide.

The compound with the shortest alkyl chain linking the TPP^+^ moiety to the **BA** scaffold, **BA-C_4_-TPP^+^Br^−^**, was the most promising, with IC_50_ values below 1 μM at 48 and 72 h of treatment. Additionally, in vitro experiments demonstrated a strong concentration-dependent uncoupling effect in mammary carcinoma cells for **BA-C_4_-TPP^+^Br^−^**. Interestingly, **BA-C_5_-TPP^+^Br^−^** and **BA-C_6_-TPP^+^Br^−^** did not exhibit a significant uncoupling effect at the tested concentrations. Furthermore, our results indicated that **BA-C_4_-TPP^+^Br^−^** induced programmed cell death via the apoptotic pathway in COLO205 cells. Moreover, in silico computation of the predicted pharmacokinetic properties (ADME) for this series of **BA** derivatives revealed only a few violations of Lipinski’s and Veber’s criteria and predicted high oral absorption. Additionally, the structural design applied to the **BA** scaffold provides favorable characteristics for its potential application in animal models. Thus, these findings suggest that **BA-C_4_-TPP^+^Br^−^** is an attractive candidate for the development of improved analogs that could overcome the challenges associated with selectivity.

## Figures and Tables

**Table 1:** In vitro activity (IC_50_, μM ± SEM; 72 h) of betulinic acid (**BA**), compounds **BA-C_4_-TPP^+^Br^−^**, **BA-C_5_-TPP^+^Br^−^**, and **BA-C_6_-TPP^+^Br^−^** vs. normal human lung fibroblasts (MRC-5), human gastric adenocarcinoma (AGS), lung cancer (SK-MES-1), bladder carcinoma (J82), and myelocytic leukemia (HL-60) cells. Etoposide was used as a positive control of cell growth inhibition. Each concentration was tested in quadruplicate and repeated three times in separate experiments. Values are expressed as means ± SD.

Compound	IC_50_ (μM)				
	MRC-5	AGS	SK-MES-1	J82	HL-60
**BA-C_4_-TPP^+^**	0.21 ± 0.01	0.35 ± 0.02	0.32 ± 0.08	0.84 ± 0.02	0.22 ± 0.04
**BA-C_5_-TPP^+^**	0.83 ± 0.02	0.77 ± 0.08	0.47 ± 0.04	1.22 ± 0.01	0.63 ± 0.05
**BA-C_6_-TPP^+^**	1.35 ± 0.1	1.27 ± 0.02	0.68 ± 0.01	1.71 ± 0.05	0.91 ± 0.01
**BA**	56.70 ± 4.3	14.12 ± 0.5	44.83 ± 2.7	32.81 ± 1.7	21.92 ± 1.5
**Etoposide**	3.96 ± 0.21	0.46 ± 0.04	2.61 ± 0.01	2.85 ± 0.02	0.82 ± 0.01

**Table 2:** In vitro activity (IC_50_, μM ± SD 24, 48, and 72 h) of betulinic acid (**BA**), **BA-C_4_-TPP^+^Br^-^**, **BA-C_5_-TPP^+^Br^−^**, and **BA-C_6_-TPP^+^Br^−^** vs. human colorectal carcinoma (HCT-15 and COLO205) cells. Each concentration was tested in quadruplicate and repeated three times in separate experiments. Values are expressed as means ± S.D.

Compound	IC_50_ (μM)					
	HCT-15			COLO205		
	24 h	48 h	72 h	24 h	48 h	72 h
**BA-C_4_-TPP^+^Br^−^**	1.46 ± 0.17	0.61 ± 0.16	0.52 ± 0.12	0.88 ± 0.22	0.45 ± 0.09	0.13 ± 0.01
**BA-C_5_-TPP^+^Br^−^**	5.17 ± 1.32	1.93 ± 0.36	1.93 ± 0.32	4.32 ± 2.4	1.62 ± 1.15	1.61 ± 0.02
**BA-C_6_-TPP^+^Br^−^**	9.04 ± 2.8	2.89 ± 0.24	2.4 ± 0.13	4.87 ± 1.43	0.84 ± 0.39	0.97 ± 0.29
**BA**	43.83 ± 3.64	9.47 ± 3.1	3.89 ± 1.3	97.68 ± 1.02	74.6 ± 2.87	34.34 ± 2.58

**Table 3:** In vitro activity (IC_50_, μM ± SEM; 24 and 48 h) of betulinic acid (**BA**), compounds **BA-C_4_-TPP^+^Br^−^**, **BA-C_5_-TPP^+^Br^−^**, and **BA-C_6_-TPP^+^Br^−^** vs. human mammary carcinoma (MCF-7, MDA-MB-231, and AU565) cells. Each concentration was tested in quadruplicate and repeated three times in separate experiments. Values are expressed as means ± S.D.

Compound	IC_50_ (μM)					
	MCF-7		MDA-MB-231		AU565	
	24 h	48 h	24 h	48 h	24 h	48 h
**BA-C_4_-TPP^+^Br^−^**	3.88 ± 0.45	0.95 ± 0.34	1.70 ± 0.44	1.01 ± 0.09	1.66 ± 0.12	0.60 ± 0.05
**BA-C_5_-TPP^+^Br^−^**	8.68 ± 1.27	1.77 ± 0.40	5.50 ± 0.60	2.53 ± 0.65	5.91 ± 0.08	1.02 ± 0.18
**BA-C_6_-TPP^+^Br^−^**	7.69 ± 1.32	4.62 ± 0.53	7.28 ± 0.53	2.66 ± 0.08	6.37 ± 0.31	1.33 ± 0.12
**BA**	-	-	-	-	33.09 ± 0.17	9.29 ± 0.98

**Table 4:** Pharmacokinetics: physicochemical properties, Lipinski’s rule of five, and Veber’s rule.

	Physicochemical Properties		Lipinski’s Rule				Veber’s Rule		
Molecule	log P ^b^	Log S ^c^	MW ^d^	MlogP ^e^	∑HBD ^f^	∑O + N ^g^	TPSA ^a^	Rotatable bonds	% ABS ^h^
**BA**	6.14	−7.71	456.70	5.82	2	3	57.53	2	89.2
**4**	6.79	−8.76	584.83	5.87	2	6	100.9	7	74.2
**BA-C_4_-TPP^+^Br^−^**	9.22	−13.85	854.01	9.06	1	3	60.12	11	88.3
**BA-C_5_-TPP^+^Br^−^**	9.52	−14.09	868.01	9.21	1	3	60.12	12	88.3
**BA-C_6_-TPP^+^Br^−^**	9.74	−13.33	882.04	9.36	1	3	60.12	13	88.3

^a^ Topological polar surface area (Å^2^); ^b^ Consensus Log P_o/w_ ^c^ Log solubility (ESOL); ^d^ molecular weight (g/mol); ^e^ Log P_o/w_; ^f^ hydrogen bond donors; ^g^ hydrogen bond acceptors. ^h^ Percentage absorption calculated as % ABS = 109 − 0.345 TPSA.

## Data Availability

The data presented in this study are available in this article. Further inquiries can be directed to the corresponding authors.
